# Endoscopic stent versus diverting stoma as a bridge to surgery for obstructive colorectal cancer: a systematic review and meta-analysis

**DOI:** 10.1007/s00423-022-02517-5

**Published:** 2022-06-06

**Authors:** Jianhao Zhang, Hong Zhu, Wenming Yang, Xueting Liu, Dechun Zhang, Xiaolian Jiang, Lie Yang, Zongguang Zhou

**Affiliations:** 1 Department of Gastrointestinal Surgery, West China Hospital of Sichuan University, No. 37 Guoxue Lane, Chengdu, 610041 Sichuan China; 2 West China School of Nursing, Sichuan University/Nursing Department, West China Hospital of Sichuan University, No. 37 Guoxue Lane, Chengdu, 610041 Sichuan China; 3 Department of Gastrointestinal Surgery, the People’s Hospital of Pengzhou, Chengdu, 611930 Sichuan China

**Keywords:** Bridge to surgery, Obstructive colorectal cancer, SEMS, Stoma

## Abstract

**Background:**

Self-expandable metallic stent (SEMS), an alternative to diverting stoma (DS), has been used as a “bridge to surgery” (BTS) to decompress acute obstruction of colorectal cancer (CRC) for decades. However, whether SEMS is a safe technique for obstruction of CRC without compromising the long-term survival of patients remains unidentified compared to those of DS. The aim of the present study was to elucidate the safety and survival outcomes of SEMS and DS.

**Methods:**

Embase, PubMed, and Medline were searched for qualified studies published until October, 2020, in which SEMS or DS was performed as a BTS without resection at the same stage. The last search was on December 5th, 2020. The Newcastle–Ottawa scale (NOS) was used to assess the quality of included studies. The major complication rate, mortality, 3-year overall survival (OS), and permanent stoma rate were estimated as outcomes.

**Results:**

The present study was registered on INPLASY (No. 2020100079). Seven eligible studies were included, involving 646 and 712 patients who underwent SEMS and DS treatments, respectively. The Clavien-Dindo I/II grade complication rate was significantly lower in the SEMS group than in the DS group (8.68 vs. 16.85%; RR, 0.59; 95% confidence interval (CI) 0.41–0.84; *P* = 0.004). The Clavien-Dindo III/IV grade complication rate was comparable in two groups (7.69 vs. 8.79%; RR, 0.82; 95% CI 0.54–1.27; *P* = 0.37). There were no statistical differences in the short-term mortality (5.16 vs. 4.53%; RR, 1.25; 95% CI 0.75–2.08; *P* = 0.39), 3-year OS (71.91 vs. 76.60%; RR, 0.93; 95% CI 0.86–1.01; *P* = 0.10), and permanent stoma rate (22.08 vs. 27.54%; RR, 0.84; 95% CI 0.67–1.06; *P* = 0.14) between the two groups.

**Conclusions:**

To some extent, SEMS is a safe BTS technique for acute obstructive CRC, without significant adverse effect on the survival of patients. Given the advantage of minimal invasion, SEMS may be a better alternative to DS for obstructive CRC. However, the conclusions remain to be discussed because of lacking high-quality randomized controlled trails.

**Supplementary Information:**

The online version contains supplementary material available at 10.1007/s00423-022-02517-5.

## Introduction

Colorectal cancer (CRC), the third most commonly diagnosed cancer, has caused an overwhelming public health and financial burden worldwide [1]. Approximately 8–29% of advanced CRC patients present with an acute obstruction, for which emergency surgery is a conventional decompression treatment [2]. For right-sided CRC, one-stage radical resection and anastomosis is the standard procedure [3]. For left-sided CRC, the risk of anastomotic leak after one-stage radical resection is relatively high because the proximal bowel is severely distended by feces [3, 4]. Therefore, Hartmann’s operation is usually performed in these patients [5]. In addition, a simple diverting stoma (DS) as a “bridge to surgery” (BTS) without concurrent tumor resection is also a common choice. Existing studies have revealed that postoperative morbidity and mortality would increase if resection is performed in an emergency care setting [6–8]. In contrast, a simple diverting colostomy as BTS is a better option because of its lower perioperative risk, shorter operation time, and quicker recovery to subsequent chemotherapy after II-stage resection surgery if necessary [9]. However, DS still has the disadvantage of requiring additional operations and developing stoma-related complications.

Self-expandable metallic stents (SEMSs) have been used as an alternative BTS technique for obstructive CRC since the 1990s [10], overcoming the limitations of DS to some extent with the advantages of minimal invasion, optimization of patient’s condition, and adequate preoperative assessment [11–13]. Some studies indeed have shown lower morbidity, mortality, and long-term stoma rates in patients who underwent SEMS treatment compared to those who underwent DS [14, 15]. However, concerns have been expressed regarding the effect of colonic stenting on the incidence of perforation and on the long-term survival in potentially curable CRC patients [4, 10, 16]. Some studies have reported relatively high rates of SEMS-related perforation, which doubts the safety of SEMS. Moreover, oncological concern for tumor cell dissemination and locoregional recurrence attributed to tumor manipulation also hamper the clinical application of SEMS [17].

However, strong evidences are needed to support the implementation of SEMS as a routine BTS. It is of great importance to conduct a systematic literature review for gastroenterologists and endoscopic surgeons to determine the best bridging strategy. Therefore, we performed a meta-analysis directly comparing SEMS with DS as a BTS for obstructive CRC with regard to both short- and long-term outcomes. To our knowledge, this is the first systematic review that directly compares SEMS and DS as a BTS for obstructive CRC.

## Materials and methods

The meta-analysis was conducted according to the Cochrane guidelines and Preferred Reporting Items for Systematic Reviews and Meta-Analyses (PRISMA) 2020 Checklist (Supplementary Material 1) [18]. There is no restriction on ethical rationality due to the nature of systematic review and meta-analysis. The present meta-analysis was registered using INPLASY (Registration number: INPLASY2020100079; https://inplasy.com/inplasy-2020-10-0079).

### Literature search

The electronic literature search was performed using PubMed, MEDLINE, EMBASE, the Cochrane Library, International Clinical Trials Registry Platform, and Google Scholar databases to identify relevant studies published until October, 2020. To ensure that all studies meeting our inclusion criteria were retrieved, three authors (Zhang, Yang, and Liu) carried out the literature and review article search. The literature search was performed under a defined search strategy (Supplementary Material 2), and the complete manuscripts of all relevant studies were searched. Additionally, reference articles were searched to identify other potentially eligible papers. The first and last search was performed on July 20th, 2020, and December 5th, 2020, respectively. The online search identified a total of 437 articles, of which seven were included in the current review. Of these, six were retrospective and one was prospective.

### Inclusion criteria

Studies meeting the following criteria were considered eligible: [1] randomized controlled trials (RCTs) or non-randomized controlled studies on stents versus stoma as BTS in patients with malignant large-bowel obstruction; (DS in the present study refers to a simple diverting ostomy without concurrent large bowel resection or cancer resection. After decompression and general condition preparation, the radical cancer resection will be performed at the next stage.) [2] patients who were planned to undergo selective radical resection surgery instead of palliative therapy; [3] analysis of two BTS techniques and at least one pre-selected outcome; [4] published in English; [5] Newcastle–Ottawa scale (NOS) scores ≥ 6.

### Exclusion criteria

Studies reporting the following were excluded: [1] benign large bowel obstruction, [2] inadequate data on defined outcomes of the two procedures, [3] original data cannot be extracted or analyzed, [4] data were impossible to calculate from the published results, [5] obstruction caused by cancers other than primary CRC, and [6] reviews, meta-analyses, case reports, meeting abstracts, letters, and unpublished articles.

### Outcome measures

The primary outcomes are as follows: [1] Major complications refer to BTS- and resection-related adverse events within 90 days after surgery, including perforation, stent dysfunction, stent migration, anastomotic leakage, stomal necrosis, prolapse, parastomal hernia, abdominal wall abscesses, and anastomotic stenoses. Non-procedure-related complications are excluded such as pulmonary infection and deep venous thrombosis; [2] Short-term mortality, death induced by BTS procedures within 90 days. The secondary outcomes were 3-year overall survival (OS) and permanent stoma rate.

In addition, definable complications were graded according to the classification of surgical complications by Clavien-Dindo. I/II grade complications refer to those with or without the need for pharmacological or invasive treatment, including blood transfusions, and total parenteral nutrition, for example, stomal necrosis, prolapse, parastomal hernia, and abdominal wall abscesses. I/II grade complications refer to those requiring surgical or endoscopic intervention or threatening life, for example, bowel perforation, stent migration, and anastomotic stenoses.

### Data extraction

Two reviewers (Zhang and Yang) independently extracted data using a standardized form after full-text assessment. They summarized the data based on the following characteristics: the first author’s name, year of publication, country, case number of each group, treatment for patients, patient demographics, clinicopathological parameters, TNM stage, neoadjuvant or adjuvant therapy, 3-year OS, major complication, short-term mortality, permanent stoma rate, and additional interventions. Any disagreement between the two reviewers on the extracted data was resolved following consultation with the third reviewer (Xueting Liu).

### Assessment of methodological quality

To assess the risk of bias and methodological quality of the cohort studies, the NOS was used to score them from 0 (worst) to 9 (best) [19]. The NOS evaluates studies with a semi-quantitative star-level system. Each included study was assessed based on the following three aspects: case selection (0–4), comparability (0–2), and outcome (0–3). Six main methods were evaluated: grouping, blinding, intention-to-treat analysis, baseline, diagnostic criteria, and control of mixed factors. Each eligible study was scored out of a maximum of 9 stars. Studies with an NOS score of 0–3, 4–6, and 7–9 were regarded as low, intermediate, and high quality, respectively. Articles that scored ≥ 6 were qualified. The quality of the included prospective study was assessed using the Cochrane’s risk bias assessment tool [20]. Even this procedure was completed by three authors (Zhang, Yang, and Liu) independently.

### Certainty assessment

JH Zhang and XT Liu will assess overall quality of evidence for every outcome independently using the five Grading of Recommendations, Assessment, Development, and Evaluations (GRADE) considerations. Direct evidence from RCTs begins at high quality, while observational study begins at low. However, the overall quality will be analyzed on five down-grade considerations (study limitations, consistency of effect, imprecision, indirectness, and publication bias) and three up-grade considerations (large magnitude of effect, dose–response relation, and plausible confounders or biases). Finally, assess the certainty of evidence as four grades: high, moderate, low, and very low.

### Statistical analysis

In this study, all analyses were performed according to the original treatment allocation method and were conducted using Review Manager (version 5.3; Nordic Cochrane Center, Cochrane, Copenhagen, Denmark). Statistical analysis was performed for a meta-analysis of proportions and pairwise comparison. We used pooled risk ratios (RRs) for all outcomes in the SEMS and DS groups to evaluate the correlation between the two BTS techniques and their outcomes. The Cochran Mantel–Haenszel method was used to combine the summary statistics, and heterogeneity was estimated using the *I*^2^ statistic, which required a fixed-effects model. Higher *I*^2^ values indicated increased heterogeneity, and *I*^2^ values > 50% indicated significant heterogeneity. Differences were considered statistically significant at *P* < 0.05. Data that could not be combined for reasons such as differences in units and measurement standards were discarded. Moreover, a subgroup analysis was performed to explore the sources of heterogeneity. Publication bias was assessed using a funnel plot and, if present, would be tested to further explore the stability of the results. A sensitivity analysis was also conducted to estimate the stability of the results.

## Results

### Study characteristics

**Fig. 1:**
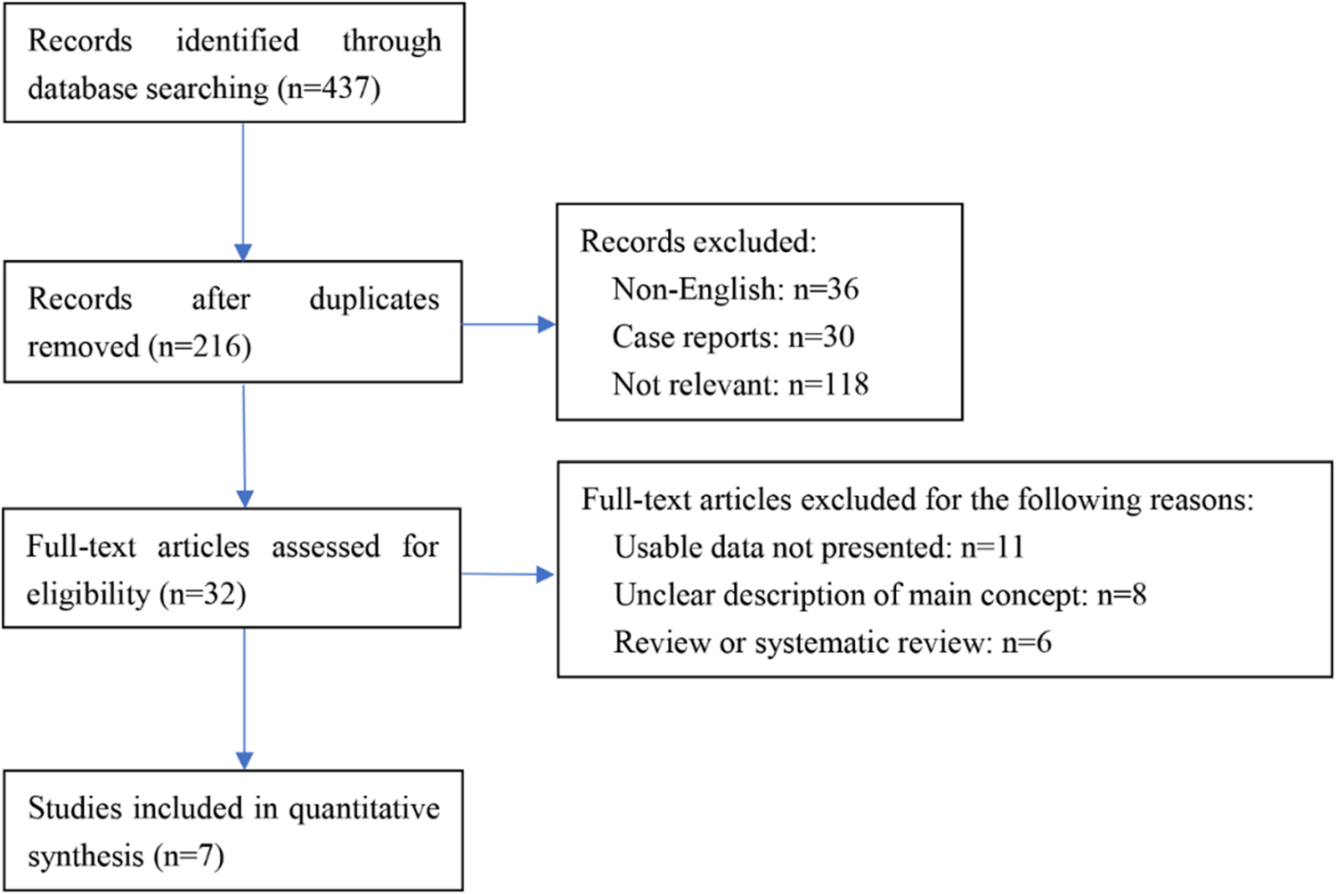
Flow chart of the systematic review

Figure 1 shows the flowchart of the study selection procedure. A total of 437 potentially relevant articles were retrieved from the initial search, with 216 remaining after duplicate removal. There were three RCT studies that met the inclusion criteria but failed to be included into analysis because the data could not be extracted or analyzed [21–23]. Seven studies involving 1358 patients were included in the current review [9, 15, 24–28]. This included six retrospective studies [9, 15, 25–28] and one prospective study [24], published in the past 6 years (2015–2020). The general characteristics of the studies are summarized in Table 1. A total of 646 and 712 patients with malignant large bowel obstruction were included in the SEMS and DS groups, respectively.

**Table 1:** General characteristics of included studies

Reference	Country	Recruitment	Treatment protocols	Type of publication	NOS scores
Veld et al. 2020 [9]	Netherlands	Jan 2009 to Dec 2016	SEMS versus DS	Retrospective	8
Mege et al. 2019 [15]	France	Jan 2000 to Dec 2015	SEMS versus DS	Retrospective	8
Tanis et al. 2015 [24]	Netherlands	Jan 2009 to Dec 2012	Emergency resection versus SEMS versus DS	Prospective	8
Amelung et al. 2016* [25]	Netherlands	Jan 2004 to Dec 2013	SEMS versus DS	Retrospective	7
Amelung et al. 2016** [26]	Netherlands	Jan 2009 to Dec 2013	Emergency resection versus SEMS versus DS	Retrospective	7
Jung et al. 2020 [27]	Korea	Feb 2016 to Aug 2018	SEMS versus DS	Retrospective	6
Öistämö et al. 2016 [28]	Sweden	Jan 1997 to Dec 2013	Emergency resection versus SEMS versus DS	Retrospective	7

*SEMS*, self-expandable metallic stent; *DS*, diverting stoma; *NOS*, Newcastle–Ottawa scale

^*^, **Two independent studies by the same first author

While six of the seven included studies were retrospectively designed [9, 15, 25–28], the NOS scores of them were at least 6, suggesting that they were all moderate or high-quality studies. Subgroup and sensitivity analyses revealed no heterogeneity (*I*^2^ = 0) across studies in the analysis of major complications, 3-year OS, and mortality. However, the heterogeneity in the analysis of permanent stoma rate was relatively higher (*I*^2^ = 17%) but regarded as mild statistically. According to the results of hypothesis tests, we found that all included studies met the requirements of comparability in age, gender, proportion of patients in IV-stage, tumor location, and the rate of adjuvant chemotherapy. None of the seven studies excluded the stage IV cases, yet accounted for relatively low and statistically comparable proportions between studies. Underlining the nature of SEMS and DS as BTS procedures, all enrolled patients were scheduled to undergo elective radical resection with curative intent, including those in stage IV. Additionally, the comparability of rate of adjuvant chemotherapy reduced confounding factors in the analysis of OS. In the study by Femke J. Amelung [25], adjuvant chemotherapy rates in the two groups differed widely (19.6% in the SEMS group vs. 54.1% in the DS group), but the difference was not statistically significant (*P* = 0.052). Moreover, there were two large-sample-based studies which used propensity score matching [9, 15] and one prospective study [24], which further improved the comparability between the groups. Given the above findings, the quality of our research and the reliability of our conclusions are guaranteed.

### Risk of bias of included studies

As all included studies were cohort studies, the quality of the enrolled studies was assessed by the NOS. The risk of related bias in the included studies was at low level (Tables 1 and 2). Sensitivity analysis did not find statistical heterogeneity among the studies for all outcomes. Regarding publication bias, funnel plots and Egger’s regression were used for meta-analyses, and there was no significant publication bias in the outcome. Certainty of the evidence of every outcome is presented in Table 3.

**Table 2:** Baseline characteristics of included studies

	Number of patients	Group	Sex (male/female)	Age (mean ± SD or range)	ASA grade (1–2/ > 3)	Tumor location (left-sided colon/right-sided colon/rectum)	pN stage (negative/positive)	M stage (0/1)	Proportion of IV-stage (%)	Adjuvant therapy (yes/no)	Proportion of taking adjuvant therapy (%)	Selective resection surgery (yes/no)	Follow-up (month)	
Veld et al. 2020 [9]	443	SEMS:203	115/88	70.5 ± 11.7	155/487 l	203/0/0	101/102	187/16	7.88	91/112	44.83	Yes	31.0	
		DS:240	149/91	68.5 ± 11.4	187/53	240/0/0	95/145	224/16	6.67	93/147	38.75	Yes	35.5	
Mege et al. 2019 [15]	518	SEMS:191	95/96	72 ± 14	103/71	186/0/5	NA	147/44	23.04	101/90	53.88	Yes	> 18	
		DS:327	185/142	71 ± 15	214/92	112/0/15	NA	276/49	14.98	173/154	52.91	Yes	> 18	
Tanis et al. 2015 [24]	331	SEMS:196 DS:135	119/77 76/59	71 (34–92) 68 (29–91)	142/51 103/32	196/0/0 135/0/0	95/96 63/65	124/45 85/32	22.96 23.70	74/117 44/89	37.76 32.59	Yes Yes	NA NA	
Amelung et al. 2016* [25]	88	SEMS:51	25/26	71.8	46/5	51/0/0	26/24	47/4	7.84	17/34	33.33	Yes	> 18	
		DS:37	14/23	66.63	36/1	37/0/0	17/20	33/4	10.81	20/17	54.05	Yes	> 18	
Amelung et al. 2016** [26]	86	SEMS:44	20/24	69.9 (53–86)	31/13	0/38/6	23/21	31/13	29.55	12/32	27.27	Yes	36	
		DS:42	24/18	64.9 (35–89)	36/6	0/26/16	15/27	27/15	35.71	16/26	38.10	Yes	29	
Jung et al. 2020 [27]	50	SEMS:23	15/8	70 (48–84)	19/4	21/0/2	9/14	18/4	17.39	15/8	65.22	Yes	12.3	
		DS:27	12/15	69 (41–88)	16/11	17/0/10	11/16	13/11	40.74	18/9	66.67	Yes	12.3	
Öistämö et al. 2016 [28]	43	SEMS:20	7/13	71 ± 10	13/7	19/0/1	9/11	NA	NA	4/15	20.00	Yes	NA	
		DS:23	13/10	67 ± 12	14/9	22/0/1	12/11	NA	NA	10/13	43.48	Yes	NA	

*SEMS*, self-expandable metallic stent; *DS*, diverting stoma; *SD*, standard deviation; *ASA*, American society of anesthesiologist; *NA*, not available

^*^, **Two independent studies by the same first author

**Table 3:** Summary of outcome measures

Outcome measures	Number of cases		Pooled estimate: mean%		Pooled RR	95% CI	*P*-value	*I*^2^ (%)	Certainty of the evidence (GRADE)
	SEMS	DS	SEMS	DS					
I/II grade complication [9, 15, 26–28]			8.68	16.85	0.59	0.41–0.84	0.004	35	Moderate
III/IV grade complication [9, 15, 26–28]	403	546	7.69	8.79	0.82	0.54–1.27	0.37	0	Low
Overall grade complication [9, 15, 26–28]			16.38	28.57	0.61	0.47–0.79	0.00001	0	Low
Short-term mortality [9, 15, 24–26]	601	662	5.16	4.53	1.25	0.75–2.08	0.39	0	Low
3-year OS [9, 15, 25]	356	470	71.91	76.6	0.93	0.86–1.01	0.1	0	Low
Permanent stoma rate [9, 15, 25]	385	512	22.08	27.54	0.84	0.67–1.06	0.14	17	Moderate

*SEMS*, self-expandable metallic stent; *DS*, diverting stoma; *RR*, risk ratio; *CI*, confidence intervals

### Primary outcomes

**Fig. 2:**
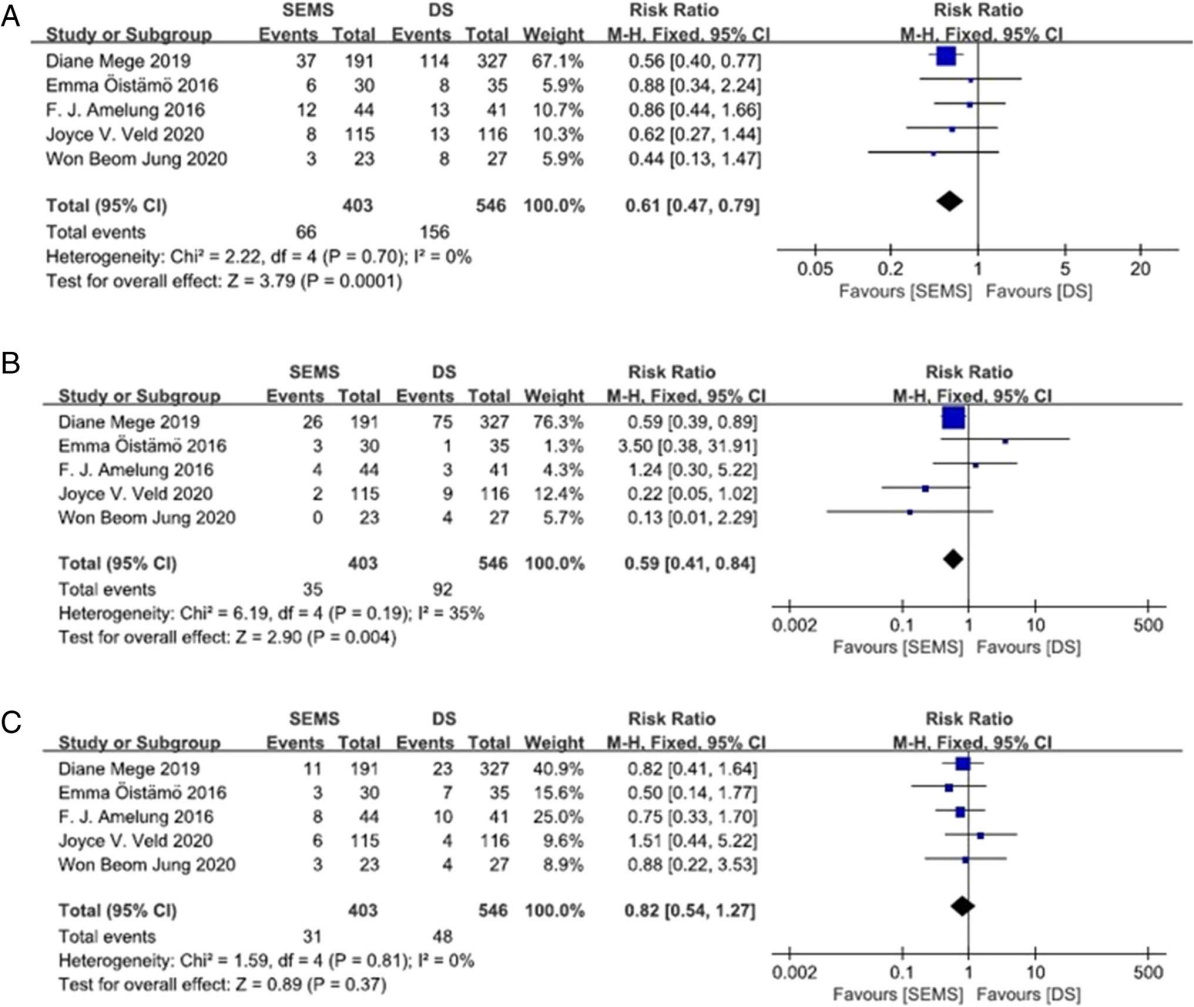
Forest plot showing risk ratio (RR) in complication rate in two BTS groups. **A** for overall complications; **B** for I/II grade complications; **C** for II/III grade complications

Complications were reported in five studies [9, 15, 26–28], with 403 patients in the SEMS group and 546 in the DS group. The overall rate of morbidity was 16.38% in the SEMS group and 28.57% in the DS group. A fixed-effects model was used, and no heterogeneity was observed. The pooled RR was 0.61 (95% CI 0.47–0.79; *P* = 0.00001). No notable publication bias was detected in the funnel plots. The Clavien-Dindo I/II grade complication rate was significantly lower in the SEMS group than in the DS group (8.68 vs. 16.85%). The Clavien-Dindo III/IV grade complication rate was comparable in two groups (7.69 vs. 8.79%). Statistically, stent insertion showed a great advantage compared to DS (Fig. 2).

**Fig. 3:**
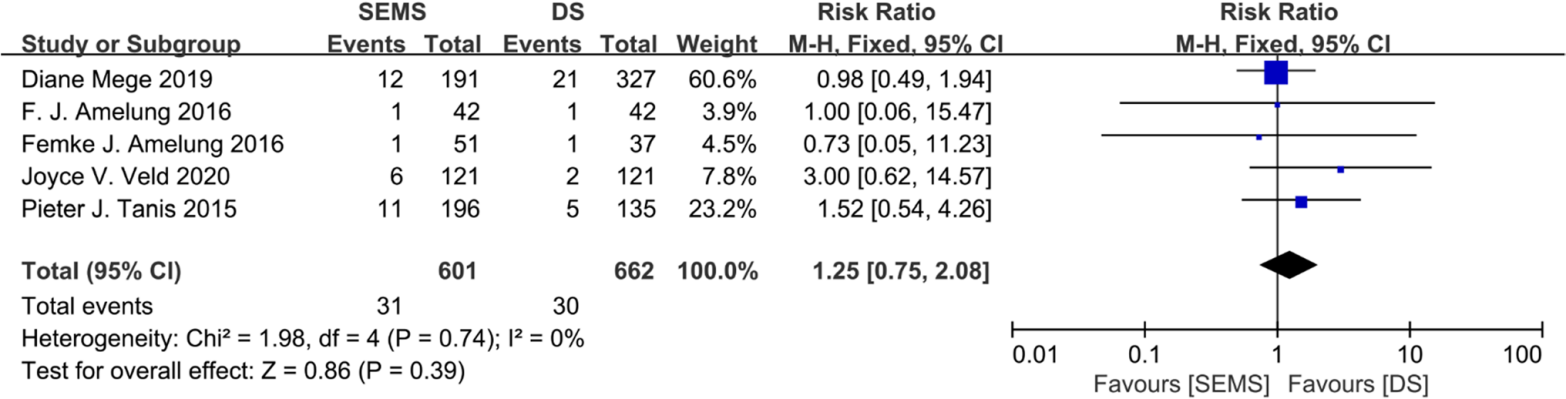
Forest plot showing risk ratio (RR) in short-term mortality in two BTS groups

Based on the different follow-up periods among the included studies, we considered short-term mortality, particularly mortality within 90 days, as an endpoint. This outcome was reported in five studies [9, 15, 24–26], with 601 patients in the SEMS group and 662 in the DS group. The short-term mortality rate was 5.16% in the SEMS group and 4.53% in the DS group, with no decrease in heterogeneity (*I*^2^ = 0). The overall RR was 1.25 (95% CI 0.75–2.08; *P* = 0.39), and there was no clear evidence of publication bias. Based on the results of our analysis, we concluded that there was a difference in the primary outcomes between the two groups (Fig. 3).

### Secondary outcomes

**Fig. 4:**
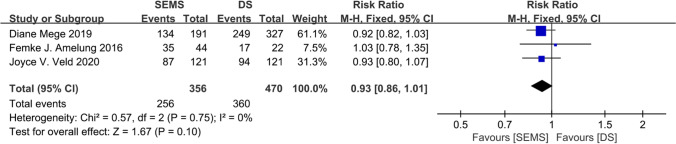
Forest plot showing risk ratio (RR) in 3-year overall survival (OS) in two BTS groups

The present meta-analysis selected 3-year OS and permanent stoma rates as secondary outcomes. The 3-year OS was reported in three studies [9, 15, 25], with 356 patients in the SEMS group and 470 in the DS group. The 3-year OS rate was 71.91% in the SEMS group and 76.60% in the DS group. No heterogeneity was observed. The fixed-effects model was used, which showed an overall RR of 0.93 (95% CI 0.86–1.01; *P* = 0.10). The 3-year OS did not differ significantly between the groups, and the funnel plot did not reveal any publication bias (Fig. 4).

**Fig. 5:**
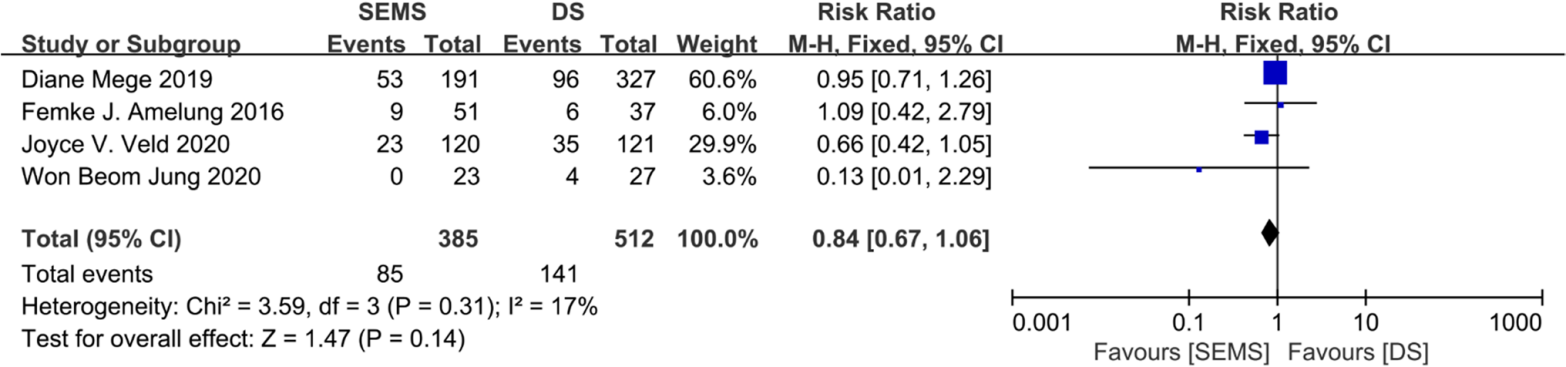
Forest plot showing risk ratio (RR) in permanent stoma rate in two BTS groups

The permanent stoma rate was reported in four studies [9, 15, 25, 27], with 385 patients in the SEMS group and 512 in the DS group. The permanent stoma rate was 22.08% in the SEMS group and 27.54% in the DS group, with an overall RR of 0.84 (95% CI 0.67–1.06; *P* = 0.14). The heterogeneity was low (*P* = 0.31, *I*^2^ = 17%) but within the permissible range. Regarding BTS, the two procedures showed no difference in the permanent stoma rate (Fig. 5). The summary of pooled outcome measures and RRs is shown in Table 3.

## Discussion

SEMS has the advantage of minimally invasive relief of obstruction, but its safety and long-term oncological outcomes remain unidentified [9, 14, 29]. The present meta-analysis included seven qualified studies comparing SEMS with DS as a bridge to resection surgery [9, 15, 24–28]. The results showed that SEMS was superior to DS in reducing the major complication rate, while not compromising short-term mortality, 3-year OS, and permanent stoma rate.

To evaluate the safety of SEMS treatment, we analyzed the major complication rate and mortality between the groups. It has been reported that the major complications of the SEMS group were bowel perforation and stent dysfunction, including technical or clinical placement failure, overgrowth, and stent migration [6, 9, 25]. As for DS, the major complications included stomal necrosis, prolapse, parastomal hernia, abdominal wall abscesses, and anastomotic stenoses. In this meta-analysis, we analyzed the complications mentioned above and found that the overall major complication rate was 16.38% in the SEMS group, which was significantly lower than that in the DS group (28.57%). For Clavien-Dindo I/II grade complication, it was 8.68% in the SEMS group, which was significantly lower than that of DS group (16.85%). For more severe Clavien-Dindo III/IV grade complication, there was no significant difference, thereby favoring endoscopic treatment.

Although the overall complication rate of SEMS treatment was lower than that of DS, the consequences of SEMS-related perforation were relatively severe. Perforation can cause acute peritonitis and sepsis, and may even be life-threatening [16, 30]. Some authors also considered the possibility of its association with negative oncological outcomes, such as dissemination of cancer cells [9, 29, 31]. In some early studies, the situation was not very optimistic, and relatively high perforation rates even contributed to the premature closure of two Dutch Stent-in II trials [14, 32]. Concern regarding both the incidence and fatality of perforation is an essential safety factor that limits the clinical application of SEMS. Our research answered the former aspect of concern. In contrast to some published studies, the overall perforation rate in our review was 6.26% and was lower than 5.0% in most of the included studies. Only two studies observed a relatively high perforation rate, reported as 8.0% in the study by Veld et al. [9] and 11.0% in the study by Diane Mege et al. [15]. The reasons for this difference may be multifactorial: One, stent-related perforation could be avoided to a great extent by comprehensively measuring the condition of patients, including the length of obstruction and stage of tumor. Two, the threshold for endoscopic surgeons to perform SEMS is relatively high. In experienced centers, perforation rates tend to be lower, and the outcomes are successful [33]. Three, endoscopic equipment has been undergoing continuous improvement. Better evaluation of stent mechanical properties would further reduce the risk of perforation [34]. Some recent studies have reported that the technical and clinical success rates of SEMS have reached 96% and 92%, respectively [35].

To clarify the latter aspect of concern that perforation would increase short-term mortality, we conducted a subsequent analysis. We found that the short-term mortality of the SEMS group did not increase significantly compared with that of the DS group. According to eligible studies [9, 15, 24–26], the short-term mortality was 5.16% in the SEMS group and 4.53% in the DS group, without any statistically significant difference. Perforation was not the primary cause of mortality in our study. The sole perforation case in a study by Femke J. Amelung [25] was reported as death due to peritonitis, and other studies did not specifically report the number of perforation-induced deaths. The existing mortality cases were supposed to be explained by the confounding of cases in stage IV and high American Society of Anesthesiology (ASA) scores. Our results are also in line with some published studies, such as the study by Sebastian et al., which reported a stent-related mortality of 0.58%. Thus, we can conclude that the safety of SEMS may not be inferior to that of conventional DS.

However, whether SEMS adversely affects the long-term oncological outcomes of potentially curable CRC remains unclear. Some authors argue that forceful expansion of the tumor during stent insertion could introduce cancer cells into ambient vessels and facilitate hematological tumor dissemination [9, 17, 23, 36, 37]. Some studies also reported higher rates of lymph node and perineural invasion after SEMS, which are used as important prognostic factors [15, 38, 39]. In contrast, some studies have reported that the OS was comparable and significantly better in the SEMS group. Our analysis revealed no association with decreased survival in the SEMS group, based on comparable oncological staging and medical treatment. The 3-year OS was 71.91% in patients undergoing SEMS treatment and 76.60% in those undergoing DS. No statistically significant difference was observed. Among the three studies we analyzed, the one by Diane Mege et al. reported worse 3-year OS (*P* = 0.0461), but their disease-free survival in the SEMS group was not worse than that in the DS group after adjustment for the propensity score. In a study by Veld et al., the rate of locoregional recurrence was also reported as no variance (15.5% of SEMS vs. 10.2% of DS), showing a low risk of tumor dissemination due to SEMS manipulation. Our results were further confirmed by a recent population-based study on SEMS versus emergency resection and colostomy, reporting a 3-year locoregional recurrence rate of 17.9 versus 11.0%, 3-year OS rate of 61.1 versus 75.1%, and 3-year disease-free survival rate of 48.5 versus 59.6%, respectively, without any significant statistical difference between the SEMS and non-SEMS groups [40].

Additionally, statistical analysis showed a comparable permanent stoma rate between the SEMS and DS group (22.08 vs. 27.54%). Many patients prefer SEMS treatment to avoid permanent stoma formation [41]; however, there is no evidence to support the conclusion that SEMS treatment could decrease the permanent stoma rate. However, patients undergoing SEMS treatment seemed to experience less postoperative pain and additional interventions. Considering the reasonable decompression effect and success rate of SEMS treatment, the risk of permanent stoma formation is low, as reported by the included studies. In contrast, patients undergoing DS treatment had more post-resection stomas and required additional interventions, such as stoma reversal and hernia repair [5].

It is noteworthy that SEMS may not be feasible in all cases. All perforation-promoting conditions should be considered as relative contraindications of SEMS, for example, severe intestinal edema and excessively long obstructed bowel. Moreover, the insertion of SEMS should be caution when the technique of endoscopy is not fully guaranteed.

To our knowledge, this is the first systematic review that directly compares SEMS and DS as a BTS for obstructive CRC; however, there are still several limitations. Firstly, there was no adequate large-sample-size and no high-quality RCTs on SEMS versus DS available. Since the interventions were beyond the control of researchers in retrospective studies, this meta-analysis inevitably confronts the problem of lacking a strong level of evidence. Secondly, publication bias in the analysis of OS was more challenging due to the inclusion of only three studies, and hence, it was difficult to assess funnel plot symmetry. Thirdly, most of the included studies did not report accurate time from BTS to resection. Only two studies mentioned that, and high heterogeneity existed [26, 28]. Furthermore, as the number of right-sided CRC cases was low, it was hard to state the impact of tumor location on safety and survival. However, considering the insufficiency of related studies, analyzing their data is far from ideal but unavoidable.

## Conclusion

SEMS, as a promising endoscopic treatment approach, may significantly reduce the overall complications compared to DS, especially Clavien-Dindo III/IV grade complication, while it does not increase the short-term mortality. The long-term prognosis of patients undergoing SEMS treatment was also not significantly worse than that of patients undergoing DS. Furthermore, SEMS does not significantly increase the rate of permanent stoma formation and may decrease the number of additional interventions. Based on these findings, we recommend that when the requisite expertise is available and contraindications are excluded, SEMS should be considered as the first-line treatment strategy for acute malignant bowel obstruction. Future research should focus on establishing definite treatment indications, optimizing endoscopic techniques, and innovating new devices to further advance SEMS.

## Supplementary Information

Below is the link to the electronic supplementary material.

Supplementary file1 (DOCX 71 KB)

Supplementary file2 (DOCX 19 KB)

## Data Availability

All data generated or analyzed and software used during this study are included in this published article.

## Declarations

### Permission

All figures, tables, or text passages in this manuscript have not been published elsewhere. All authors agreed to submit this manuscript for publication.
