# Supplementary material for: Endoscopic stent versus diverting stoma as a bridge to surgery for obstructive colorectal cancer: a systematic review and meta-analysis
Source: Langenbecks Arch Surg. 2022 Jun 6;407(8):3275–85. doi: 10.1007/s00423-022-02517-5 (PMC9722815; doi:10.1007/s00423-022-02517-5)
Supplement: Supplementary file 2 — Supplementary file2 (DOCX 19 KB) [file 423_2022_2517_MOESM2_ESM.docx]

Literature Search Strategy

1. Colorectal neoplasms/ Colonic neoplasms/ Rectal neoplasms/

2. (colon* or colorect* or intestin* or sigmoid* or rect* or bowel or malignan* or acute)/

3. (carcinom* or neoplas* or adenocarcinom* or cancer* or tumor* or tumour* or adenom* or malignan*)/

4. (obstruction or obstructed or obstructing or stenoses or stenosis or stricture) /

5. (stoma or stomas or stomata or stomal or parastom* or colostomy or decompression) /

6. (colectomy or colectomies or sigmoidectomy or sigmoidectomies) /

7. (stent* or SEM or SEMS or SEMT or SEMTs or 'Bridge to surgery') /

8. Exp Intestinal Obstruction/ colostomy/ surgical stomas/ Self expandable metallic stents/ Stents

9. Limit to English/ Humans not animals
